# Antithrombotic strategy following valve-in-valve transcatheter aortic valve replacement. A German Statutory Health Claims data analysis

**DOI:** 10.1007/s00392-025-02635-2

**Published:** 2025-03-20

**Authors:** Sebastian Heyne, Christopher Hohmann, Sascha Macherey-Meyer, Max M. Meertens, Elmar Kuhn, Ursula Marschall, Hendrik Wienemann, Victor Mauri, Matti Adam, Stephan Baldus, Samuel Lee

**Affiliations:** 1 Clinic III for Internal Medicine, Faculty of Medicine, University Hospital Cologne, University of Cologne, Kerpener Str. 62, 50937 Cologne, Germany; 2 Department of Cardiac Surgery, Faculty of Medicine, University Hospital Cologne, University of Cologne, Cologne, Germany; 3 BARMER, Wuppertal, Germany

**Keywords:** ViV-TAVR, Antiplatelet, Anticoagulation, DOAC, VKA

## Abstract

**Aims:**

Valve-in-valve transcatheter aortic valve replacement (ViV-TAVR) procedures are increasingly used. Specific recommendations on antithrombotic strategies following ViV-TAVR are lacking. We aimed to assess the efficacy of different antithrombotic strategies following ViV-TAVR.

**Methods and results:**

We performed a retrospective analysis of German Statutory Health Claims data following ViV-TAVR stratified by antithrombotic strategies according to prescription within 90 days. Antithrombotic regimens included antiplatelet therapy (APT), direct oral anticoagulants (DOACs) or vitamin K antagonists (VKAs). The composite endpoint was all-cause mortality, stroke and/or systemic embolism (SSE) and mechanical complication of heart valve prosthesis at 12 months. Cox proportional hazard regression models were used to compare outcomes. In total, 908 patients between 2005 and 2022 were identified. Of these, 286 received DOACs, 99 received VKAs, 351 received APT exclusively and 172 had no prescription. The incidence of the composite endpoint was 20.8% in the APT group, 20.3% in the DOAC group and 25.3% in the VKA group which was not statistically significantly different. The rate of SSE in the acetylsalicylic acid (ASA) mono group was higher compared to the dual antiplatelet therapy (DAPT) group (27.3% vs. 12.4%, univariable HR 0.42, 95% CI [0.19, 0.95], *p* = 0.03).

**Conclusion:**

In this analysis of German Health Claims data, DOACs seemed to be a safe alternative to VKAs and APT. ASA monotherapy was associated with higher rates of SSE compared to DAPT. Given the high risk of bias of this retrospective analysis and the growing use of valve-in-valve procedures, randomized controlled trials are needed to confirm these findings.

**Supplementary Information:**

The online version contains supplementary material available at 10.1007/s00392-025-02635-2.

## Introduction

In recent years, the indication for transcatheter aortic valve replacement (TAVR) has been expanded to younger, lower-risk patients supported by the results of randomized controlled trials [1–4].With the more frequent use of TAVR in younger patients, lifetime management becomes increasingly important [5]. Valve-in-valve transcatheter aortic valve replacement (ViV-TAVR) offers an alternative to conventional aortic valve replacement to treat not only degenerated TAVR but also surgically implanted bioprosthetic valves [6].

However, ViV-TAVR is associated with a higher risk of leaflet thrombosis and valve deterioration [7–9].Therefore, optimal antithrombotic treatment is crucial. The current European guidelines recommend lifelong single antiplatelet therapy (SAPT) after TAVR or the continuation of oral anticoagulation (OAC) when there is a concomitant indication for OAC like atrial fibrillation (AF) [1]. American guidelines recommend lifelong SAPT and consider temporary dual antiplatelet therapy (DAPT) or OAC with a vitamin-K antagonist (VKA) for up to 6 months [2].Due to a lack of explicit data, no specific recommendations for antithrombotic therapy after ViV-TAVR exist. Only a few ViV-TAVR patients have been assessed as part of larger TAVR trials like ATLANTIS and GALILEO with conflicting results [10, 11]. Although apixaban reduced leaflet thrombosis compared to DAPT in patients without an indication for oral anticoagulation in the ATLANTIS trial, there was no difference in clinical outcomes between both strategies. Noteworthy, all-cause mortality was numerically but not statistically significantly higher in the apixaban group compared to DAPT. In contrast, a rivaroxaban based antithrombotic strategy was associated with a higher risk of thromboembolic events or death compared to an antiplatelet only strategy in patients without indication for oral anticoagulation in the GALILEO trial.

Given the higher risk of leaflet thrombosis in ViV-TAVR and the limited available data, it remains unclear whether the existing guideline recommendations for TAVR are applicable for ViV-TAVR. Therefore, the aim of this retrospective analysis of longitudinal German Statuary Health claims data was to assess different real-world antithrombotic strategies after ViV-TAVR and their impact on short- to midterm clinical effectiveness and safety outcomes.

## Methods

This retrospective cohort study was based on anonymized data from the BARMER Data Warehouse. Therefore, ethics approval was not required and the European General Data Protection Regulation did not apply. BARMER is the second largest German statutory health insurance provider covering approximately 8.7 million individuals. The BARMER Data Warehouse provides longitudinal information about patient demographics as well as in- and outpatient healthcare services between 2005 and 2022. These include discharge dates, and diagnoses via International Classification of Diseases (ICD) codes (Tenth Revision, German Modification) and operation and procedure classification system (OPS) codes (German modification of International Classification of Procedures in Medicine, ICPM). Additionally, the database provides information on drug prescriptions including the date of prescription based on Anatomical Therapeutic Chemical (ATC) codes. The corresponding ICD, OPS and ATC codes used in this analysis are available in the supplementary appendix.

### Patients and antithrombotic therapy

We identified adult patients (age ≥ 18 years) with either two separate TAVR OPS codes on different days or a TAVR OPS code following an OPS code for surgical implantation of a bioprosthetic aortic valve. The date of the second procedure was the index date. Baseline characteristics of patients were assessed according to main and secondary discharge diagnoses and confirmed ambulatory diagnoses prior to the index date. Antithrombotic therapy was identified using ATC codes of prescriptions following ViV-TAVR (see supplementary appendix for detailed allocation process). Based on the prescription data, patients were stratified into three distinct groups: Antiplatelet therapy (APT) with any single or dual antiplatelet therapy but without OAC, OAC with DOACs irrespective of concomitant antiplatelet therapy and OAC with VKAs irrespective of concomitant antiplatelet therapy. Additionally, a subgroup analysis of patients with and without an indication for OAC was performed. Lastly, a subgroup analysis of patients with acetylsalicylic acid (ASA) monotherapy and DAPT with ASA and clopidogrel was performed in the APT group.

### Clinical endpoints

The efficacy endpoint included death from any cause, ischemic stroke and/or systemic embolism (SSE) and mechanical complication of heart valve prosthesis (see supplementary appendix for detailed ICD codes). Since the ICD-10 code for mechanical complication of heart valve prosthesis also codes for (para-)valvular leakage and displacement of the heart valve prosthesis which are usually diagnosed within the index hospitalization but not attributable to the antithrombotic therapy, this ICD 10-code was censored for the first 15 days after the index procedure.

The safety endpoint was intracranial, extracranial or gastrointestinal bleeding. Intracranial bleeding was defined as subarachnoidal, intracerebral and other non-traumatic or traumatic intracranial bleeding. Extracranial bleeding was defined as bleeding with anemia, hemothorax, conjunctival or retinal hemorrhage, unspecified, recurrent and persistent hematuria, hemorrhage from respiratory passages, hemarthrosis and other abnormal uterine or vaginal bleeding (see supplementary appendix for detailed ICD codes).

### Statistical methods

Baseline categorical variables are presented as percentages while continuous variables are presented as mean with standard deviation. Treatment effects were calculated by univariable and multivariable Cox proportional-hazard regression models using age, AF, chronic kidney disease, congestive heart failure, and concomitant APT as covariables for the main analyses. Subgroups were only analyzed using univariable Cox proportional-hazard regression models due to smaller group sizes. Statistical significance was assessed by the log-rank test. Hazard ratios (HR) and corresponding 95% confidence intervals (CI) with cumulative incidence curves are given for the primary and secondary endpoints and subgroup analyses. Secondary outcomes were the individual components of the composite endpoint. All patients were censored at 1 year for the main analyses. We additionally performed exploratory analyses with 5 years of follow-up and in patients with the index procedure before 2015 and since 2015. All analyses were performed with R (version 4.3.1).

## Results

### Baseline characteristics

In total, 908 patients were identified that received ViV-TAVR between 2005 and 2022. Of these, 172 had no documented prescription for any antithrombotic therapy. These were excluded from further quantitative analysis. Of the remaining patients, 286 (38.9%) received DOACs, 99 (13.5%) received VKAs and 351 (47.7%) received APT. In the DOAC group, apixaban was the most common DOAC (60.5%) followed by rivaroxaban (22.7%). Approximately half of the DOAC patients (51.0%) received concomitant APT. In the VKA group all but one patient received phenprocoumon. The other patient received warfarin. Of the VKA patients, 47.5% of patients had a concomitant prescription for APT. In the APT group, 51.0% of patients had a prescription for ASA and 88.3% of patients had a prescription for clopidogrel. Subsequently, 41.6% had a prescription for dual antiplatelet therapy. The mean age was 77.4 ± 8.4 years, 79.4 ± 6.5 years and 78.4 ± 6.4 years for the APT, DOAC and VKA groups, respectively. Atrial fibrillation or flutter was more common in patients receiving DOACs (88.1%) or VKAs (80.8%) compared to patients receiving APT (42.7%). Congestive heart failure was also more common in patients receiving DOACs (41.6%) and VKAs (40.4%) compared to patients receiving APT (28.8%). Baseline characteristics are summarized in Table 1.

**Table 1:** Baseline characteristics of all patients

	Any APT (*n* = 351)	Any DOAC (*n* = 286)	Any VKA (*n* = 99)
Demographics			
Age [years]	77.4 ± 8.4	79.4 ± 6.5	78.4 ± 6.4
Sex [male, %]	42.5%	44.1%	48.5%
Initial procedure			
TAVR	19.4%	18.2%	19.2%
SAVR	80.6%	81.8%	80.8%
Medical history			
Arterial Hypertension [%]	98.0%	100%	98.9%
Diabetes [%]	50.4%	46.2%	56.6%
Dyslipidemia [%]	92.9%	90.9%	93.9%
Adipositas [%]	53.3%	52.8%	54.6%
Coronary artery disease [%]	90.0%	88.5%	91.9%
History of coronary artery bypass surgery [%]	37.3%	38.8%	43.4%
History of percutaneous coronary intervention [%]	30.2%	27.9%	28.3%
History of myocardial infarction [%]	23.9%	22.0%	24.2%
Congestive heart failure [%]	28.8%	41.6%	40.0%
Atrial fibrillation/flutter [%]	42.7%	88.1%	80.8%
CHA_2_DS_2_-VASc-Score	5.2 ± 1.5	5.5 ± 1.4	5.4 ± 1.4
Modified HAS-BLED-Score	4.3 ± 0.9	4.4 ± 0.9	4.5 ± 0.8
History of Stroke [%]	13.7%	17.8%	15.2%
Chronic obstructive pulmonary disease [%]	35.9%	37.8%	31.3%
Chronic kidney disease [%]	51.9%	57.7%	65.7%
Reduced liver function [%]	4.6%	3.5%	4.0%
History of bleeding [%]	69.5%	69.9%	75.8%
History of venous thromboembolism [%]	7.1%	9.1%	6.1%
History of/Active Cancer [%]	41.0%	50.0%	46.5%
Medical therapy			
Antiplatelets			
ASA	51.0%	6.6%	8.1%
Clopidogrel	88.3%	47.6%	40.4%
Ticagrelor	2.3%	0%	1.0%
Prasugrel	0%	0%	0%
Dual antiplatelet therapy	41.6%	3.2%	2.0%
Direct oral anticoagulants			
Apixaban	–	60.5%	–
Dabigatran	–	4.9%	–
Edoxaban	–	11.9%	–
Rivaroxaban	–	22.7%	–

### Antithrombotic strategies following ViV-TAVR

**Fig. 1:**
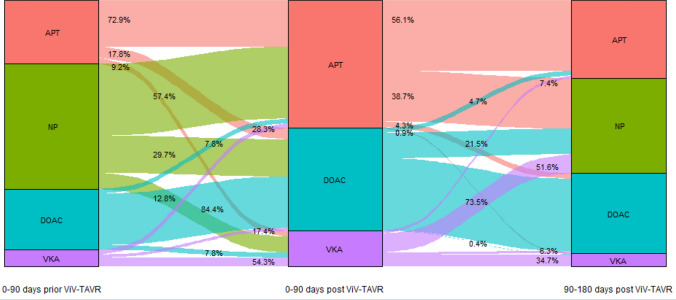
Alluvial plot of antithrombotic drug prescriptions prior to and after ViV-TAVR. *APT* antiplatelet therapy, *DOAC* direct oral anticoagulant, *NP* no prescription, *ViV-TAVR* valve-in-valve transcatheter aortic valve replacement, *VKA* vitamin K antagonist

Following ViV-TAVR, most patients received APT (47.7%), followed by DOACs (38.9%) and VKAs (13.4%). The majority continued the antithrombotic treatment they were prescribed in the 90-day period prior to ViV-TAVR. Of the patients who had no prescriptions for antithrombotic therapy prior to the index procedure, most patients were prescribed APT (57.4%) after ViV-TAVR followed by DOACs (29.7%) and VKAs (12.8%). In the VKA group, 51.6% of patients had no follow-up prescription for antithrombotic treatment three months after the procedure. In the APT and DOAC groups, 38.7% and 21.5% of patients had no follow-up prescription respectively. Only a few patients were switched from DOAC to VKA or vice versa und only approximately 5% of patients were switched from APT to OAK with DOAC or VKA after three months. Figure 1 summarizes the prescriptions patterns and changes prior to and after ViV-TAVR.

### Efficacy endpoints

**Fig. 2:**
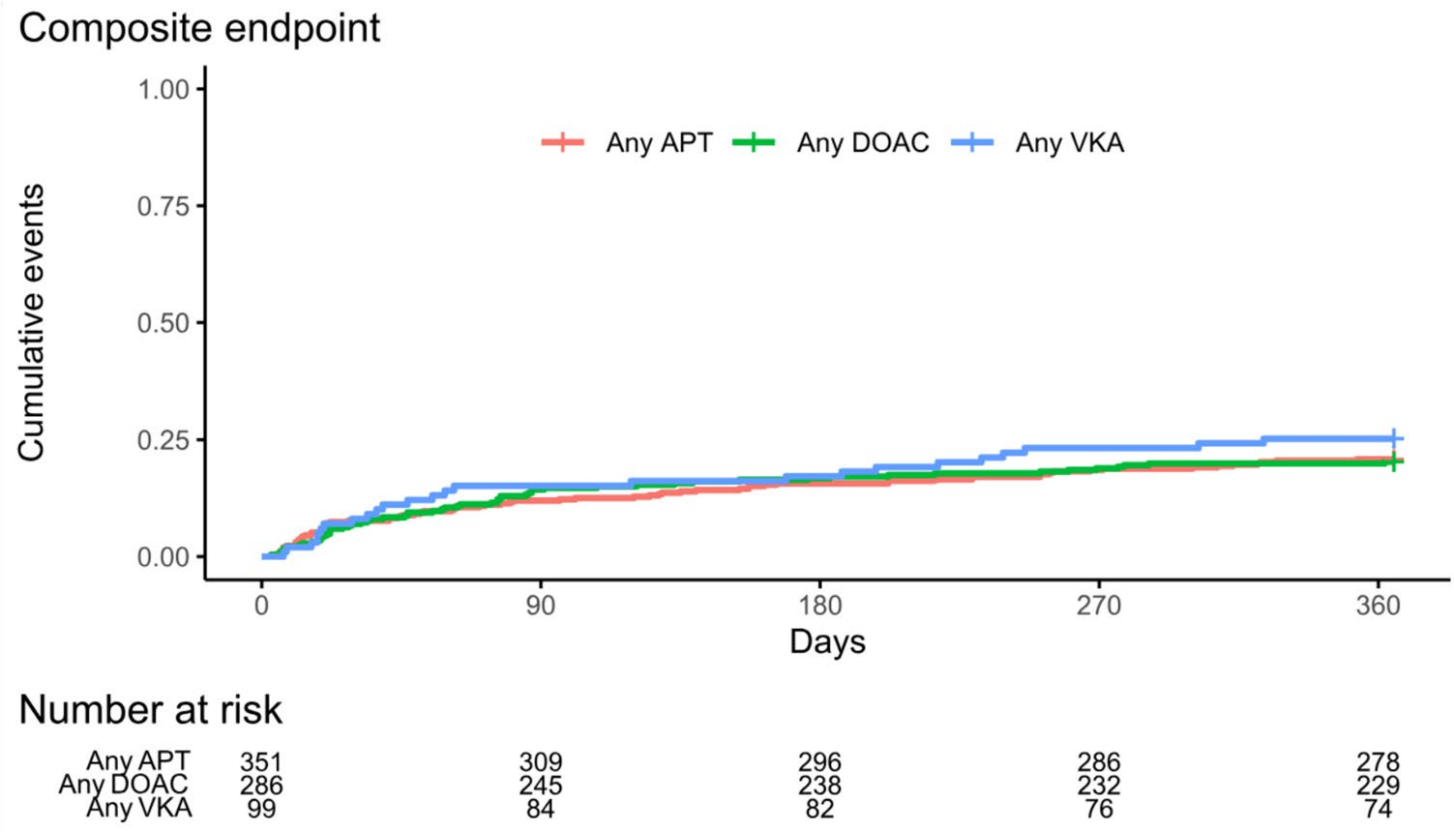
Kaplan–Meier-plot of cumulative incidence all-cause mortality, stroke and/or systemic embolism and mechanical complication of heart valve prosthesis

The incidence of the composite endpoint of all-cause mortality, SSE and mechanical complication of heart valve prosthesis at 1 year was 20.8% in the APT group, 20.3% in the DOAC group and 25.3% in the VKA group (Fig. 2). There was no statistically significant difference between groups in both the univariable and multivariable analyses (Table 2).

**Table 2:** Results of the univariable and multivariable Cox proportional-hazard regression model analyses

	% (n/N)			APT vs DOAC			APT vs VKA			DOAC vs. VKA		
	APT	DOAC	VKA	Univariable HR	*p*	Multivariable HR	Univariable HR	*p*	Multivariable HR	Univariable HR	*p*	Multivariable HR
Composite Endpoint	20.8% (73/351)	20.3% (58/286)	25.3% (25/99)	0.98 (0.69, 1.38)	0.9	0.89 (0.57, 1.38)	1.24 (0.79, 1.96)	0.4	0.83 (0.46, 1.48)	1.27 (0.79, 2.03)	0.3	1.25 (0.78, 2.01)
All-cause mortality	7.9% (28/351)	8.7% (25/286)	6.1% (6/99)	1.11 (0.65, 1.91)	0.7	1.29 (0.65, 2.57)	0.76 (0.31, 1.83)	0.5	0.75 (0.26, 2.17)	0.68 (0.28, 1.67)	0.4	0.63 (0.26, 1.56)
SSE	12.8% (45/351)	9.8% (28/286)	15.2% (15/99)	0.76 (0.47, 1.22)	0.3	0.58 (0.32, 1.06)	1.11 (0.62, 1.99)	0.7	0.59 (0.28, 1.22)	1.47 (0.79, 2.76)	0.2	1.42 (0.75, 2.69)
Mechanical complication of THV	3.1% (11/351)	3.8% (11/286)	8.1% (8/99)	1.23 (0.53, 2.83)	0.6	1.13 (0.39, 3.27)	2.65 (1.07, 6.59)	0.03*	2.67 (0.83, 8.60)	2.14 (0.86, 5.31)	0.09	2.19 (0.87, 5.56)
Intra-, extracranial or gastrointestinal bleeding	35.6% (125/351)	36.7% (105/286)	39.4% (39/99)	1.05 (0.81, 1.36)	0.8	1.00 (0.72, 1.38)	1.05 (0.73, 1.51)	0.8	1.00 (0.64, 1.56)	1.04 (0.72, 1.50)	0.8	1.03 (0.71, 1.49)

There was also no difference between groups at 5 years of follow-up (supplementary Table S2 and Figure S1). Contrastingly, in patients with index procedure before 2015, patients with prescriptions for VKAs had a significantly higher incidence of the composite endpoint while in the subgroup of patients with index procedure after 2015 there was also no difference in the incidence of the cumulative endpoint between groups (supplementary Figure S2 and Tables S3 and S4). Baseline characteristics of both groups are summarized in the supplementary appendix.

**Fig. 3:**
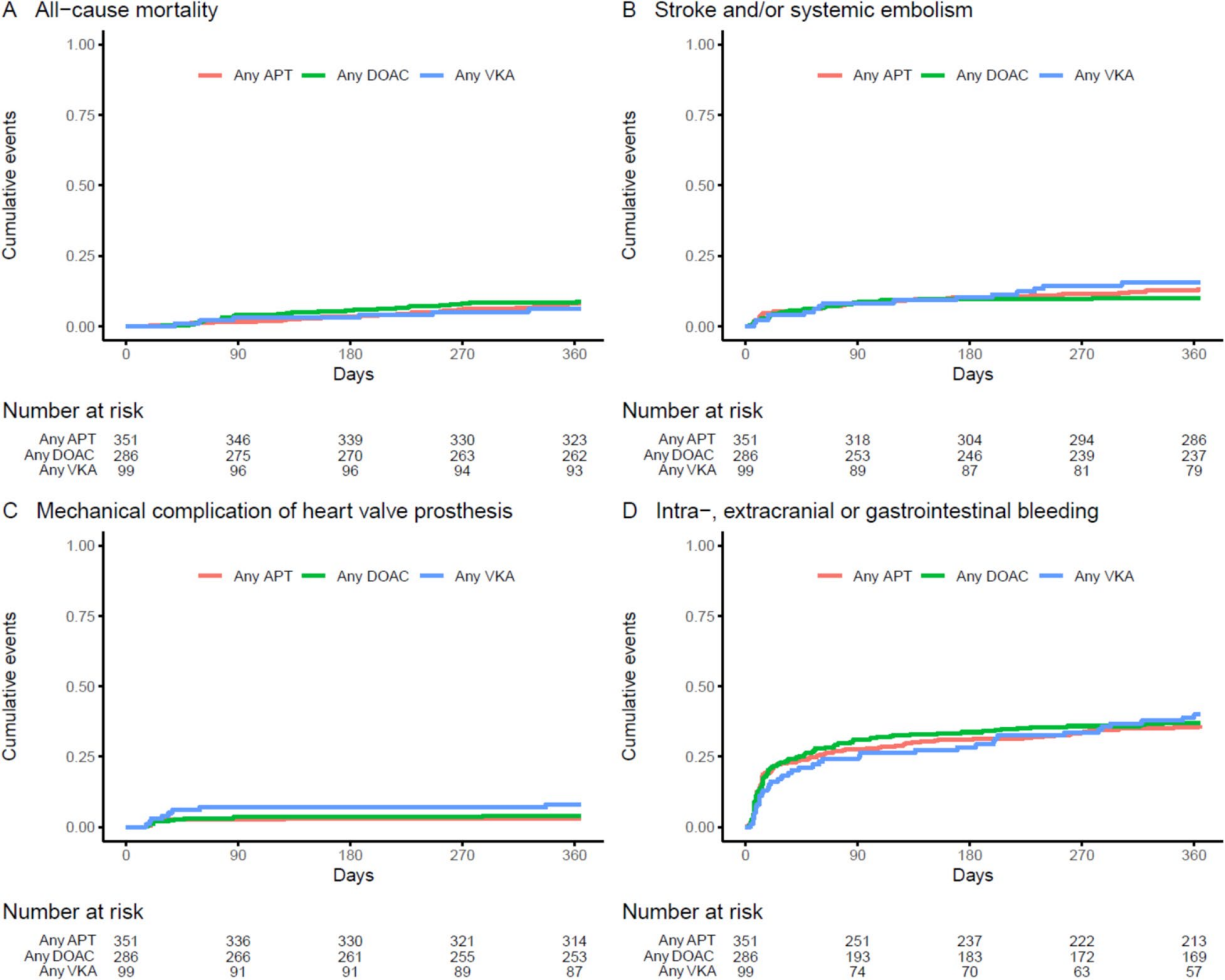
Kaplan–Meier-plots of cumulative incidence for **A** all-cause mortality, **B** stroke and/or systemic embolism, **C** mechanical complication of heart valve prosthesis and **D** intra-, extracranial or gastrointestinal bleeding

During the first year of follow-up, 7.9% died in the APT group, 8.7% in the DOAC group and 6.1% in the VKA group (Fig. 3A). These differences were not statistically significant (Table 2). Compared to the APT (12.8%) and VKA (15.2%) groups, SSE occurred numerically less often (9.8%) in the DOAC group (Fig. 3B). This effect was not statistically significant in the univariable (APT vs. DOAC: HR 0.76, 95% CI [0.47, 1.22], DOAC vs. VKA: HR 1.47, 95% CI [0.79, 2.76], Table 2) and multivariable analyses (APT vs. DOAC: HR 0.58, 95% CI [0.32, 1.06], DOAC vs. VKA: HR 1.42, 95% CI [0.75, 2.69], Table 2).

Mechanical complications of heart valve prosthesis occurred statistically significantly more frequently in the VKA group compared to the APT group (8.1% vs. 3.1%, HR 2.65, 95% CI [1.07, 6.59], *p* = 0.03, Table 2). This effect was not significant in the multivariable analysis (HR 2.67, 95% CI [0.83, 8.60], Table 2). Mechanical complications were similar in the APT group compared to the DOAC group (3.1 vs. 3.8%).

### Safety endpoints

Intra-, extracerebral or gastrointestinal bleeding occurred in 35.6% of patients in the APT group, 36.7% of patients in the DOAC group, and 39.4% of patients in the VKA group (Fig. 3D). Statistically, there were no significant differences between groups in both the univariable and multivariable analyses (Table 2).

### Subgroup analyses ASA mono vs. DAPT with ASA and clopidogrel

Supplementary Table S6 summarizes the baseline characteristics of the subgroup of patients with ASA monotherapy and DAPT with ASA and clopidogrel. 33 patients were treated with ASA mono and 137 with ASA and clopidogrel. DAPT patients were slightly younger and less often had congestive heart failure. The rate of atrial fibrillation was 39.5% in the ASA mono and 35.0% in the DAPT group.

There was a trend towards an increased rate of the composite endpoint of death, stroke and/or systemic embolism and mechanical complication of heart valve prosthesis in the ASA mono group (36.4% vs. 21.2%, univariable HR 0.53, 95% CI [0.27, 1.03], *p* = 0.06, Supplementary Table S7). This was driven by a statistically significantly higher rate of SSE in the ASA mono group (27.3% vs. 12.4%, univariable HR 0.42, 95% CI [0.19, 0.95], *p* = 0.03, Supplementary Table S7). All other endpoints were not statistically significantly different between both groups (Supplementary Table S7).

### Subgroup analyses stratified by need for oral anticoagulation

Of the patients without the need for oral anticoagulation, 201 (79.1%) patients received APT, 34 (13.4%) received DOACs and 19 (7.5%) received VKAs. Baseline characteristics for the subgroups with and without indication for OAC are summarized in Supplementary Table S8. The composite endpoint occurred numerically albeit not statistically significantly more often in the VKA group (26.3%) compared to the DOAC (17.6%) and APT (18.9%) groups (Supplementary Table S9). This was mainly driven by a numerically higher rate of mechanical complications of heart valve prosthesis (VKA 10.5% vs DOAC 2.9% vs. APT 3.0%). Of the patients with an indication for OAC, 252 (52.3%) patients received DOACs, 150 (31.1%) received APT and 80 (16.6%) received VKAs. There was a trend towards less SSE in the DOAC group compared to the APT group (9.5% vs. 16.0%, univariable HR 0.59, 95% CI [0.34, 1.05], *p* = 0.07, Supplementary Table S10). There was no difference for all other outcomes.

## Discussion

To the best of our knowledge, this is the largest analysis of antithrombotic strategies following ViV-TAVR. The findings of this retrospective German claims data analysis show that antithrombotic therapies varied substantially between patients in a real-world setting. This was to be expected given the uncertainty in evidence regarding antithrombotic strategies following ViV-TAVR in recent guidelines. Patients without an indication for OAC were primarily treated with APT while patients with an indication for OAC were mainly treated with DOACs. DOACs seemed to be a safe alternative to VKAs and APT both short- and long-term and in patients with and without an indication for oral anticoagulation. Importantly, approximately 42% of patients who were treated with APT exclusively had an indication for oral anticoagulation due to atrial fibrillation or flutter. Despite not being statistically significant, the rate of SSE was numerically higher in the APT group compared to the DOAC group which might be explained by the insufficient antithrombotic management. This hypothesis is supported by the subgroup of patients with an indication for OAC that were treated with APT who showed a numerically higher rate of SSE compared to patients without the need for OAC. Whether this finding is due to the uncertainty in guidelines regarding antithrombotic strategies in ViV-TAVR, unawareness of the diagnoses by the treating physicians, miscoding or other reasons remains unclear but warrants further investigation.

Likewise, the rate of the composite endpoint of all-cause mortality, SSE or mechanical complication of heart valve prosthesis was numerically albeit not statistically significantly higher in the VKA group compared to the APT and DOAC groups. This effect was mainly driven by a statistically significantly higher rate of mechanical complications of heart valve prostheses in the univariable analysis. It was also mainly driven by patients with the index procedure before 2015. However, only a few patients were included in the early study period. Therefore, these results should be interpreted with caution. Given the retrospective nature of this study, the results of the multivariable analyses and the findings of previous trials like the ATLANTIS trial, it is reasonable to assume that these findings are based on a selection bias in that patients with a higher risk of valve thrombosis were prescribed VKAs more often. [10] In that regard, it has to be noted that APT patients were slightly younger and had substantially lower baseline rates of congestive heart failure and chronic kidney disease compared to DOAC and VKA patients.

Finally, DAPT with ASA and clopidogrel were associated with significantly less SSE compared to ASA monotherapy. Again, the rate of atrial fibrillation was high in both groups. Therefore, it is reasonable to assume that the high rates of stroke in both groups are at least partially attributable to inadequate antithrombotic strategies. This finding is worrisome and warrants further investigation.

Several limitations have to be considered when interpreting the results of this study. Given the retrospective nature of this analysis, selection bias, as already discussed, is most likely present, especially in some of the subgroup analyses. Furthermore, health insurance claims data depend on the quality of coding of in- and outpatient diagnoses. However, data from administrative US claims data and US pivotal trials of TAVR showed a good correlation between claims data for procedural data and mortality [12]. Moreover, antithrombotic therapies were identified using ATC prescription codes. This introduced two problems: In Germany, ASA can be bought without prescription over the counter. Therefore, it is likely that the amount of patients receiving ASA was underestimated in the present analysis. One might assume that a considerable number of patients without documented prescription received ASA—considering the high prevalence of coronary artery disease. Given that patients were stratified by antithrombotic medication prescriptions, this also introduced survivorship bias, as patients had to survive until they were able to receive a respective prescription. It remains unclear how this bias might have affected the individual groups. Additionally, we had no dosing information for the respective therapies. Furthermore, approximately 40% of patients alive at 3 months following ViV-TAVR were switched to other antithrombotic medications. This questions the effect of the initial treatment strategy on long-term clinical outcomes. Lastly, it is possible that the index valve-in-valve procedure was not successful but, because of the ICD coding rules, patients would still have been included. Given that ViV-TAVR implantation success rates are reported to be very high at around 97%, this effect will however most likely be negligible [13].

## Conclusion

In this analysis of real-world German claims data, antithrombotic strategies and durations varied widely following ViV-TAVR. DOACs seemed to be a safe alternative to VKAs and APT both in patients with and without an indication for oral anticoagulation. ASA monotherapy was associated with higher rates of SSE compared to dual antiplatelet therapy. Given the high risk of bias of this retrospective analysis and the growing use of valve-in-valve procedures, randomized controlled trials are needed to confirm these findings. The high rate of patients with atrial fibrillation who were treated with antiplatelets exclusively stands in contrast to current guideline recommendations and warrants further investigation. 

## Supplementary Information

Below is the link to the electronic supplementary material.

Supplementary file1 (DOCX 659 KB)

## Data Availability

The data that support the findings of this study are not openly available due to national privacy regulations.
