# Supplementary material for: Antithrombotic strategy following valve-in-valve transcatheter aortic valve replacement. A German Statutory Health Claims data analysis
Source: Clin Res Cardiol. 2025 Mar 20;115(7):1099–106. doi: 10.1007/s00392-025-02635-2 (PMC13249617; doi:10.1007/s00392-025-02635-2)
Supplement: Supplementary file 1 — Supplementary file1 (DOCX 659 KB) [file 392_2025_2635_MOESM1_ESM.docx]

**Details of the treatment group allocation process**

1. Antithrombotic therapy was identified by prescription within 90 days of the index procedure date using ATC codes.
2. For patients that had no prescription within 90 days after the index procedure date, the intervals 90 days prior to the index procedure and up to 180 days after the index procedure were assessed under the assumption that the patients continued their previous antithrombotic therapy when there was the same prescription prior to and after the procedure.
3. Patients that did not fulfil any of the abovementioned criteria were excluded from the analysis.
4. The remaining patients were then stratified into three distinct groups: Antiplatelet therapy with any single or dual antiplatelet therapy but without OAC, OAC with DOACs irrespective of concomitant antiplatelet therapy and OAC with VKAs irrespective of concomitant antiplatelet therapy

| **ICD Codes** | |
| --- | --- |
| Atrial fibrillation/flutter | I48% |
| Diabetes | E11%, E10% |
| Adipositas | E66% |
| Chronic kidney disease | N18%, N19 |
| Reduced liver function | K70.2, K70.3%, K70.4%, K71.7, K72.1, K72.7, K72.9, K74% |
| Stroke | I63% |
| Myocardial infarction | I21%, I22% |
| Chronic coronary syndrome | I25% |
| History of coronary artery bypass grafting | Z95.1 |
| History of coronary artery stenting | Z95.5 |
| Congestive heart failure | I50.0% |
| Arterial hypertension | I15%, I10% |
| History of cancer | C%, D1%, D2%, D3%, D4% |
| History of venous thromboembolism | I82% |
| Systemic embolism | I74% |
| Mechanical complication of heart valve prosthesis | T82.0 |
| Composite Bleeding | I60%, I61%, I62%, S06.3, D62%, S06.4, S06.5, S06.6, J94.2, H11.3, H35.6, H43.1, H45.0, N02, R04, R31, R58, M25.0, N93, K250.0, K25.2, K25.4, K25.6, K26.0, K26.2, K26.4, K26.6, K27.0, K27.2, K27.4, K27.6, K28.0, K28.2, K28.4, K28.6, K29.0, I85.0, K62.5, K92.0, K92.1, K92.2 |
| **OPS codes** | |
| SAVR | 5-351.0% |
| TAVR | 5-35a.0% |
| **ATC codes** | |
| VKAs | B01AA% |
| Apixaban | B01AF02 |
| Dabigatran | B01AE07 |
| Edoxaban | B01AF03 |
| Rivaroxaban | B01AF01, B01AX06 |
| ASA | B01AC06, C07AA57, C07FX02, N02AJ18, N02BA01, N02BA51, N02BA71 |
| Clopidogrel | B01AC04 |
| Ticagrelor | B01AC24 |
| Prasugrel | B01AC22 |
| ASA/Clopidogrel | B01AC34 |

**Supplementary table S1:** ICD-, OPS- and ATC-codes used for the identification of diagnoses, procedures and antithrombotic medication. ASA = acetylic salicylic acid, ATC = anatomical therapeutic chemical, ICD = International Statistical Classification of Diseases and Related Health Problems, OPS = operation and procedure classification system

|  | % (n/N) | | | APT vs DOAC | | | APT vs VKA | | | DOAC vs. VKA | | |
| --- | --- | --- | --- | --- | --- | --- | --- | --- | --- | --- | --- | --- |
|  | APT | DOAC | VKA | Univariable  HR | p | Multivariable  HR | Univariable  HR | p | Multivariable  HR | Univariable  HR | p | Multivariable  HR |
| Composite Endpoint | 25.4%  (89/351) | 23.4%  (67/286) | 29.3%  (29/99) | 0.89  (0.69, 1.14) | 0.4 | 0.83  (0.60, 1.14) | 1.07  (0.91, 1.26) | 0.4 | 0.98  (0.79, 1.20) | 1.27  (0.91, 1.79) | 0.2 | 1.26  (0.89, 1.78) |
| All-cause mortality | 30.5%  (107/351) | 27.3%  (78/286) | 33.3%  (33/99) | 0.90  (0.67, 1.21) | 0.5 | 0.87  (0.61, 1.26) | 1.05  (0.87, 1.28) | 0.6 | 1.02  (0.80, 1.29) | 1.22  (0.81, 1.83) | 0.4 | 1.23  (0.81, 1.85) |
| SSE | 17.4%  (61/351) | 15.4%  (44/286) | 22.2%  (22/99) | 0.88  (0.59, 1.29) | 0.5 | 0.82  (0.50, 1.36) | 1.11  (0.87, 1.41) | 0.4 | 0.91  (0.67, 1.24) | 1.39  (0.84, 2.33) | 0.2 | 1.42  (0.85, 2.38) |
| Mechanical complication of THV | 3.4%  (12/351) | 4.9%  (14/286) | 10.1%  (10/99) | 1.44  (0.66, 3.11) | 0.4 | 1.56  (0.57, 4.29) | 1.75  (1.15, 2.66) | 0.006 | 1.88  (1.11, 3.18) | 2.12  (0.94, 4.77) | 0.08 | 2.07  (0.91, 4.72) |
| Intra-, extracranial or gastrointestinal bleeding | 50.1%  (176/351) | 47.9%  (137/286) | 52.5%  (52/99) | 1.01  (0.80, 1.26) | 1.0 | 0.98  (0.75, 1.29) | 1.02  (0.87, 1.12) | 0.8 | 1.05  (0.87, 1.27) | 1.07  (0.79, 1.49) | 0.6 | 1.08  (0.78, 1.49) |

**Supplementary Table S2.** Results of the univariable Cox proportional-hazard regression model with censoring at 5 years

|  | % (n/N) | | | APT vs DOAC | | APT vs. VKA | | DOAC vs VKA | |
| --- | --- | --- | --- | --- | --- | --- | --- | --- | --- |
|  | APT | DOAC | VKA | Univariable | p | Univariable | p | Univariable | p |
| Composite Endpoint | 18.9%  (10/53) | 22.2%  (2/9) | 46.2%  (6/13) | 1.12  (0.25, 5.29) | 0.8 | 1.69  (1.02, 2.80) | 0.03* | 2.59  (0.52, 12.85) | 0.2 |

**Supplementary Table S3.** Results of the univariable Cox proportional-hazard regression model subgroup analyses of patients with index procedure before 2015

|  | % (n/N) | | | APT vs DOAC | | APT vs. VKA | | DOAC vs VKA | |
| --- | --- | --- | --- | --- | --- | --- | --- | --- | --- |
|  | APT | DOAC | VKA | Univariable | p | Univariable | p | Univariable | p |
| Composite Endpoint | 21.1%  (63/298) | 20.2%  (56/277) | 22.1%  (19/86) | 0.96  (0.67, 1.37) | 0.8 | 1.02  (0.79, 1.32) | 0.9 | 1.09  (0.65, 1.85) | 0.7 |

**Supplementary Table S4.** Results of the univariable Cox proportional-hazard regression model subgroup analyses of patients with index procedure since 2015

|  | Patients with index procedure before 2015 | | | Patients with index procedure since 2015 | | |
| --- | --- | --- | --- | --- | --- | --- |
|  | APT (n=53) | DOAC (n=9) | VKA (n=13) | APT (n=298) | DOAC (n=277) | VKA (n=86) |
| **Demographics** |  |  |  |  |  |  |
| Age [Jahre; mean ± SD] | 75.3 ± 10.2 | 77.9 ± 6.4 | 73.1 ± 7.9 | 77.8 ± 7.9 | 79.4 ± 6.5 | 79.2 ± 5.8 |
| Sex [male, %] | 54.7% | 66.7% | 76.9% | 40.3% | 43.3% | 44.2% |
| **Medical history** |  |  |  |  |  |  |
| Arterial Hypertension [%] | 96.2% | 100% | 100% | 98.3% | 100% | 98.8% |
| Diabetes [%] | 60.4% | 77.8% | 53.6% | 48.7% | 45.1% | 56.9% |
| Dyslipidemia [%] | 84.9% | 88.9% | 92.31% | 94.3% | 90.9% | 94.2% |
| Adipositas [%] | 41.5% | 44.4% | 46.2% | 55.4% | 53.1% | 55.8% |
| Coronary artery disease [%] | 83.0% | 100% | 76.9% | 91.3% | 88.1% | 94.2% |
| History of coronary artery bypass surgery [%] | 33.9% | 22.2% | 46.2% | 37.92% | 39.4% | 43.0% |
| History of percutaneous coronary intervention [%] | 33.9% | 11.1% | 15.4% | 29.5% | 28.5% | 30.2% |
| History of myocardial infarction [%] | 18.9% | 44.4% | 0% | 24.8% | 21.3% | 27.9% |
| Congestive heart failure [%] | 39.6% | 33.3% | 46.2% | 26.9% | 41.9% | 39.5% |
| Atrial fibrillation/flutter [%] | 56.6% | 88.9% | 92.3% | 40.3% | 88.1% | 79.1% |
| CHA_2_DS_2_-VASc-Score | 4.9 ± 1.5 | 6.0 ± 1.2 | 4.8 ± 1.7 | 5.3 ± 1.4 | 5.4 ± 1.4 | 5.5 ± 1.3 |
| Modified HAS-BLED-Score | 4.1 ±0.9 | 4.7 ± 0.5 | 4.2 ± 0.8 | 4.3 ± 0.9 | 4.4 ± 0.9 | 4.5 ± 0.8 |
| History of Stroke [%] | 7.6% | 44.4% | 23.1% | 14.8% | 16.9% | 13.9% |
| Chronic obstructive pulmonary disease [%] | 45.3% | 44.4% | 46.2% | 34.2% | 37.6% | 29.1% |
| Chronic kidney disease [%] | 62.3% | 44.4% | 46.2% | 50.0% | 58.1% | 68.6% |
| Reduced liver function [%] | 7.6% | 11.1% | 0% | 4.0% | 3.3% | 4.7% |
| History of major bleeding [%] | 52.8% | 77.8% | 61.5% | 72.5% | 69.7% | 77.9% |
| History of venous thromboembolism [%] | 3.8% | 0% | 0% | 7.7% | 9.4% | 6.9% |
| History of/Active Cancer [%] | 45.3% | 44.4% | 61.5% | 40.3% | 50.2% | 44.2% |
| **Medical therapy** |  |  |  |  |  |  |
| **Antiplatelets** |  |  |  |  |  |  |
| ASS | 50.9% | 0% | 23.1% | 51.0% | 6.9% | 5.8% |
| Clopidogrel | 92.5% | 33.3% | 38.5% | 87.6% | 48.0% | 40.7% |
| Ticagrelor | 0% | 0% | 0% | 2.7% | 0% | 1.2% |
| Prasugrel | 0% | 0% | 0% | 0% | 0% | 0% |
| Dual antiplatelet therapy | 43.4% | 0% | 7.7% | 41.3% | 3.3% | 1.2% |
| **Direct oral anticoagulants** |  |  |  |  |  |  |
| Apixaban | - | 11.1% | - | - | 62.1% | - |
| Dabigatran | - | 11.1% | - | - | 4.7% | - |
| Edoxaban | - | 0% | - | - | 12.3% | - |
| Rivaroxaban | - | 77.8% | - | - | 20.9% | - |

**Supplementary Table S5:** Baseline characteristics of the patients in the subgroup analysis of patients with index procedure before and since 2015. ASA = acetylic salicylic acid, DAPT = dual antiplatelet therapy

|  | ASA mono | DAPT (ASA + Clopidogrel) |
| --- | --- | --- |
|  | n=33 | n=137 |
| **Demographics** |  |  |
| Age [years; mean ± SD] | 79.2 ± 6.9 | 77.6 ± 8.3 |
| Sex [male, %] | 45.5% | 44.5% |
| **Medical history** |  |  |
| Arterial Hypertension [%] | 96.97% | 99.3% |
| Diabetes [%] | 45.5% | 51.8% |
| Dyslipidemia [%] | 87.9% | 95.6% |
| Adipositas [%] | 51.5% | 62.8% |
| Coronary artery disease [%] | 81.8% | 90.5% |
| History of coronary artery bypass surgery [%] | 30.3% | 39.4% |
| History of percutaneous coronary intervention [%] | 24.2% | 39.4% |
| History of myocardial infarction [%] | 24.2% | 24.8% |
| Congestive heart failure [%] | 36.4% | 29.2% |
| Atrial fibrillation/flutter [%] | 39.45% | 35.0% |
| CHA_2_DS_2_-VASc-Score | 5.3 ± 1.4 | 5.3 ± 1.5 |
| Modified HAS-BLED-Score | 4.4 ± 0.8 | 4.3 ± 0.8 |
| History of Stroke [%] | 18.2% | 13.9% |
| Chronic obstructive pulmonary disease [%] | 33.3% | 32.9% |
| Chronic kidney disease [%] | 54.6% | 53.3% |
| Reduced liver function [%] | 0% | 0% |
| History of major bleeding [%] | 69.7% | 73.7% |
| History of venous thromboembolism [%] | 6.1% | 8.8% |
| History of/Active Cancer [%] | 48.5% | 40.9% |

**Supplementary Table S6:** Baseline characteristics of the patients in the subgroup analysis of patients treated with ASA mono or DAPT with ASA and clopidogrel. ASA = acetylic salicylic acid, DAPT = dual antiplatelet therapy

|  | % (n/N) | | ASA vs. DAPT | |
| --- | --- | --- | --- | --- |
| Endpoint | ASA | DAPT | Univariable | p |
| Composite Endpoint | 12/33  (36.4%) | 29/137  (21.2%) | 0.53  (0.27, 1.03) | 0.06 |
| All-cause mortality | 3/33  (9.1%) | 12/137  (8.8%) | 0.98  (0.28, 3.46) | 1.0 |
| Stroke and/or systemic embolism | 9/33  (27.3%) | 17/137  (12.4%) | 0.42  (0.19, 0.95) | 0.03* |
| Mechanical complication of THV | 2/33  (6.1%) | 5/137  (3.7%) | 0.60  (0.12, 3.09) | 0.5 |
| Major bleeding | 12/33  (36.4%) | 54/137  (37.4%) | 1.09  (0.58, 2.04) | 0.8 |

**Supplementary Table S7.** Results of the univariable Cox proportional-hazard regression model for the subgroup analyses of ASS mono vs. DAPT with ASS and Clopidogrel. ASA = acetylic salicylic acid, DAPT = dual antiplatelet therapy, THV = transcatheter heart valve

|  | Without indication for OAC | | | With indication for OAC | | |
| --- | --- | --- | --- | --- | --- | --- |
|  | APT (n=201) | DOAC (n=34) | VKA (n=19) | APT (n=150) | DOAC (n=252) | VKA (n=80) |
| **Demographics** |  |  |  |  |  |  |
| Age [Jahre; mean ± SD] | 77.3 ± 8.3 | 77.4 ± 8.9 | 78.4 ± 4.7 | 77.7 ± 8.5 | 79.6 ± 6.0 | 78.4 ± 6.7 |
| Sex [male, %] | 41.3% | 32.4% | 47.4% | 44.0% | 45.6% | 48.8% |
| **Medical history** |  |  |  |  |  |  |
| Arterial Hypertension [%] | 97.5% | 100% | 100% | 98.7% | 100% | 98.8% |
| Diabetes [%] | 46.3% | 29.4% | 42.1% | 56.0% | 48.4% | 60.0% |
| Dyslipidemia [%] | 92.0% | 91.2% | 94.7% | 94.0% | 90.9% | 93.8% |
| Adipositas [%] | 52.7% | 55.9% | 63.2% | 54.0% | 52.4% | 52.5% |
| Coronary artery disease [%] | 87.1% | 67.6% | 94.7% | 94.0% | 91.3% | 91.3% |
| History of coronary artery bypass surgery [%] | 39.3% | 38.2% | 52.6% | 34.7% | 39.9% | 41.3% |
| History of percutaneous coronary intervention [%] | 27.9% | 23.5% | 36.8% | 33.3% | 28.6% | 26.3% |
| History of myocardial infarction [%] | 17.9% | 14.7% | 31.6% | 32.0% | 23.0% | 22.5% |
| Congestive heart failure [%] | 25.4% | 8.8% | 21.1% | 33.3% | 46.0% | 45.0% |
| Atrial fibrillation/flutter [%] | 0% | 0% | 0% | 100% | 100% | 100% |
| CHA_2_DS_2_-VASc-Score | 5.1 ± 1.5 | 4.6 ± 1.3 | 4.9 ± 0.9 | 5.4 ± 1.4 | 5.6 ± 1.4 | 5.6 ± 1.4 |
| Modified HAS-BLED-Score | 4.1 ± 0.9 | 3.9 ± 0.9 | 4.2 ± 0.9 | 4.5 ± 0.9 | 4.5 ± 0.8 | 4.6 ± 0.8 |
| History of Stroke [%] | 11.4% | 8.8% | 5.3% | 16.7% | 19.0% | 17.5% |
| Chronic obstructive pulmonary disease [%] | 35.3% | 23.5% | 26.3% | 36.7% | 39.7% | 32.5% |
| Chronic kidney disease [%] | 43.3% | 26.5% | 42.1% | 63.3% | 61.9% | 71.3% |
| Reduced liver function [%] | 4.5% | 0% | 0% | 4.7% | 4.0% | 5.0% |
| History of major bleeding [%] | 65.2% | 61. 8% | 68.4% | 75.3% | 71.0% | 77.5% |
| History of venous thromboembolism [%] | 6.0% | 8.8% | 5.3% | 8.7% | 9.1% | 6.3% |
| History of/Active Cancer [%] | 41.8% | 47.1% | 52.6% | 40.0% | 50.4% | 45.0% |
| **Medical therapy** |  |  |  |  |  |  |
| **Antiplatelets** |  |  |  |  |  |  |
| ASS | 56.7% | 8.8% | 0% | 43.3% | 6.3% | 10.0% |
| Clopidogrel | 88.1% | 29.4% | 47.4% | 88.7% | 50.0% | 38.8% |
| Ticagrelor | 2.0% | 0% | 5.3% | 2.7% | 0% | 0% |
| Prasugrel | 0% | 0% | 0% | 0% | 0% | 0% |
| Dual antiplatelet therapy | 46.8% | 2.9% | 0% | 34.7% | 3.2% | 2.5% |
| **Direct oral anticoagulants** |  |  |  |  |  |  |
| Apixaban | - | 73.5% | - | - | 58.7% | - |
| Dabigatran | - | 2.9% | - | - | 5.2% | - |
| Edoxaban | - | 8.8% | - | - | 12.3% | - |
| Rivaroxaban | - | 14.7% | - | - | 23.8% | - |

**Supplementary table S8:** Baseline characteristics of the patients in the subgroup analysis according to indication for oral anticoagulation

|  | % (n/N) | | | APT vs DOAC | | APT vs. VKA | | DOAC vs VKA | |
| --- | --- | --- | --- | --- | --- | --- | --- | --- | --- |
|  | APT | DOAC | VKA | Univariable | p | Univariable | p | Univariable | p |
| Composite Endpoint | 18.9%  (38/201) | 17.6%  (6/34) | 26.3%  (5/19) | 0.97  (0.41, 2.31) | 1.0 | 1.49  (0.59, 3.81) | 0.4 | 1.45  (0.44, 4.7) | 0.5 |
| All-cause mortality | 8.5%  (17/201) | 5.9%  (2/34) | 10.5%  (2/19) | 0.71  (0.16, 3.06) | 0.6 | 1.30  (0.30, 5.64) | 0.7 | 1.7  (0.25, 12.4) | 0.6 |
| Stroke and/or systemic embolism | 10.4%  (21/201) | 11.8%  (4/34) | 10.5%  (2/19) | 1.11  (0.38, 3.24) | 0.8 | 1.04  (0.24, 4.45) | 1.0 | 0.85  (0.16, 4.66) | 0.9 |
| Mechanical complication of THV | 3.0%  (6/201) | 2.9%  (1/34) | 10.5%  (2/19) | 0.98  (0.12, 8.11) | 1.0 | 3.52  (0.71, 17.43) | 0.1 | 4.00  (0.36, 44.14) | 0.2 |
| Intra-, extra- or gastrointestinal bleeding | 34.3%  (69/201) | 26.5%  (9/34) | 47.4%  (9/19 | 0.63  (0.31, 1.26) | 0.2 | 1.33  (0.66, 2.67) | 0.4 | 1.89  (0.75, 4.79) | 0.2 |

**Supplementary Table S9.** Results of the univariable Cox proportional-hazard regression model subgroup analyses of patients without the need for oral anticoagulation

|  | % (n/N) | | | APT vs DOAC | | APT vs. VKA | | DOAC vs VKA | |
| --- | --- | --- | --- | --- | --- | --- | --- | --- | --- |
|  | APT | DOAC | VKA | Univariable | p | Univariable | p | Univariable | p |
| Composite Endpoint | 23.3%  (35/150) | 20.6%  (52/252) | 25.0%  (20/80) | 0.86  (0.56, 1.32) | 0.5 | 1.06  (0.61, 1.83) | 0.8 | 1.23  (0.74, 2.07) | 0.4 |
| All-cause mortality | 7.3%  (11/150) | 9.1%  (23/252) | 5.0%  (4/80) | 1.25  (0.61, 2.57) | 0.5 | 0.67  (0.21, 2.11) | 0.5 | 0.54  (0.19, 1.55) | 0.6 |
| Stroke and/or systemic embolism | 16.0%  (24/150) | 9.5%  (24/252) | 16.3%  (13/80) | 0.59  (0.34, 1.05) | 0.07 | 0.95  (0.48, 1.86) | 0.9 | 1.69  (0.86, 3.32) | 0.1 |
| Mechanical complication of THV | 3.3%  (5/150) | 4.0%  (10/252) | 7.5%  (6/80) | 1.21  (0.41, 3.53) | 0.7 | 2.31  (0.70, 7.56) | 0.2 | 1.94  (0.70, 5.32 | 0.2 |
| Intra-, extra- or gastrointestinal bleeding | 37.3%  (56/150) | 38.1%  (96/252) | 37.5%  (30/80) | 1.04  (0.75, 1.45) | 0.8 | 0.89  (0.57, 1.38) | 0.6 | 0.93  (0.62, 1.40) | 0.7 |

**Supplementary Table S10.** Results of the univariable Cox proportional-hazard regression model subgroup analyses of patients with the need for oral anticoagulation


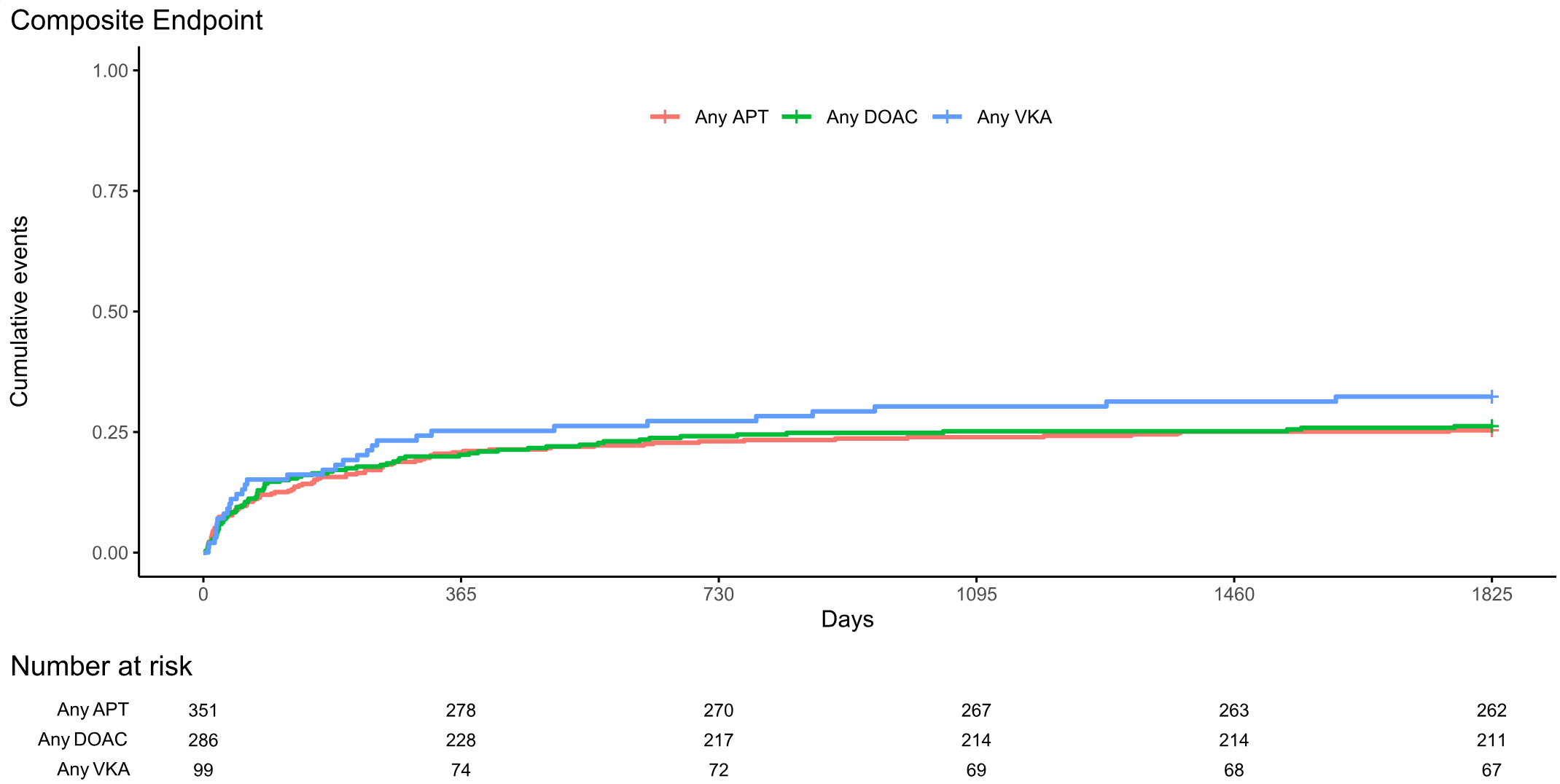


**Supplementary Figure S1** Kaplan-Meier-plot of cumulative incidence of the composite endpoint of all-cause mortality, stroke and/or systemic embolism and mechanical complication of heart valve prosthesis


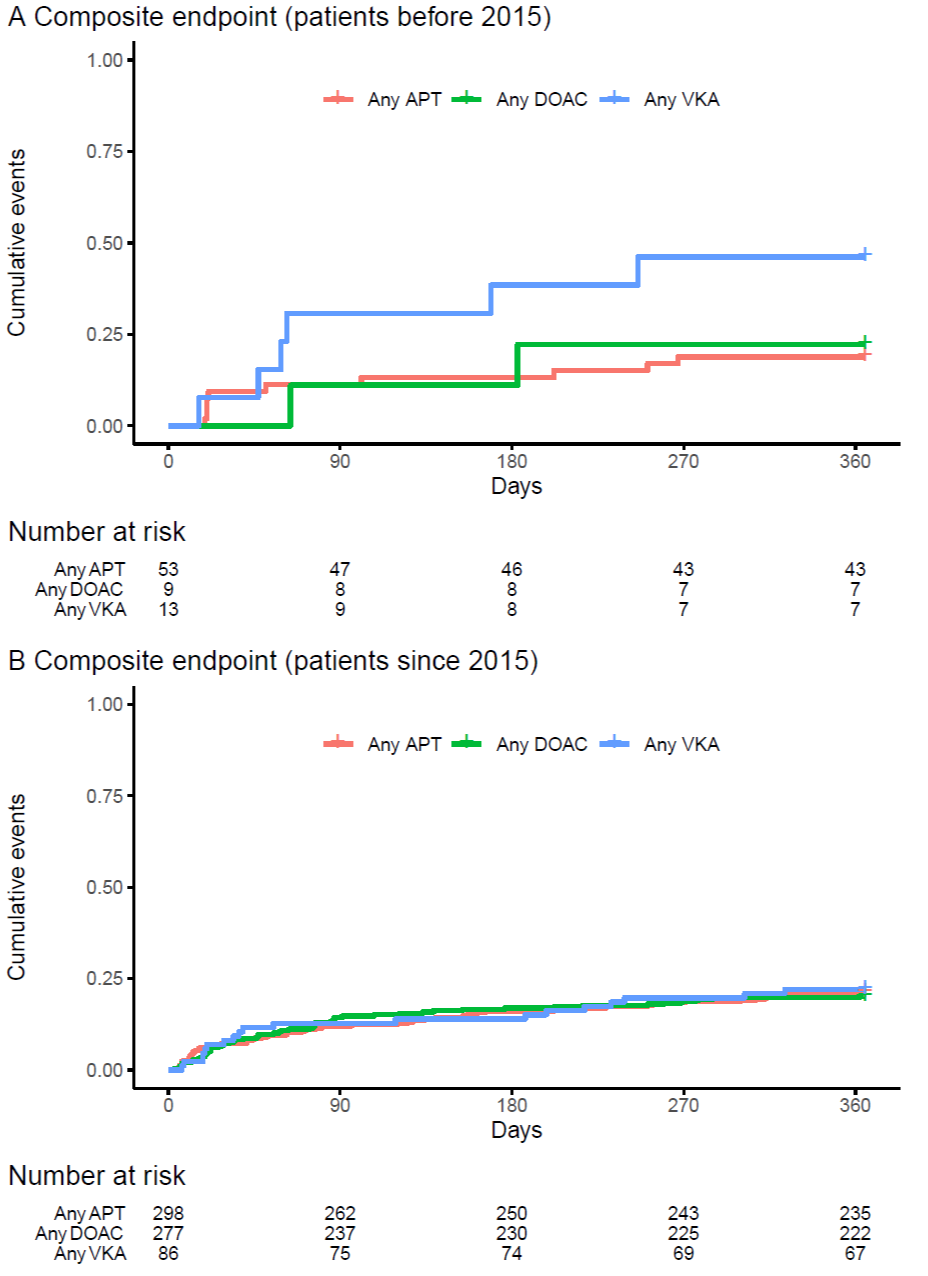


**Supplementary Figure S2** Kaplan-Meier-plots of cumulative incidence of the composite endpoint of all-cause mortality, stroke and/or systemic embolism and mechanical complication of heart valve prosthesis in patients with index procedure before 2015 (A) and since 2015 (B)


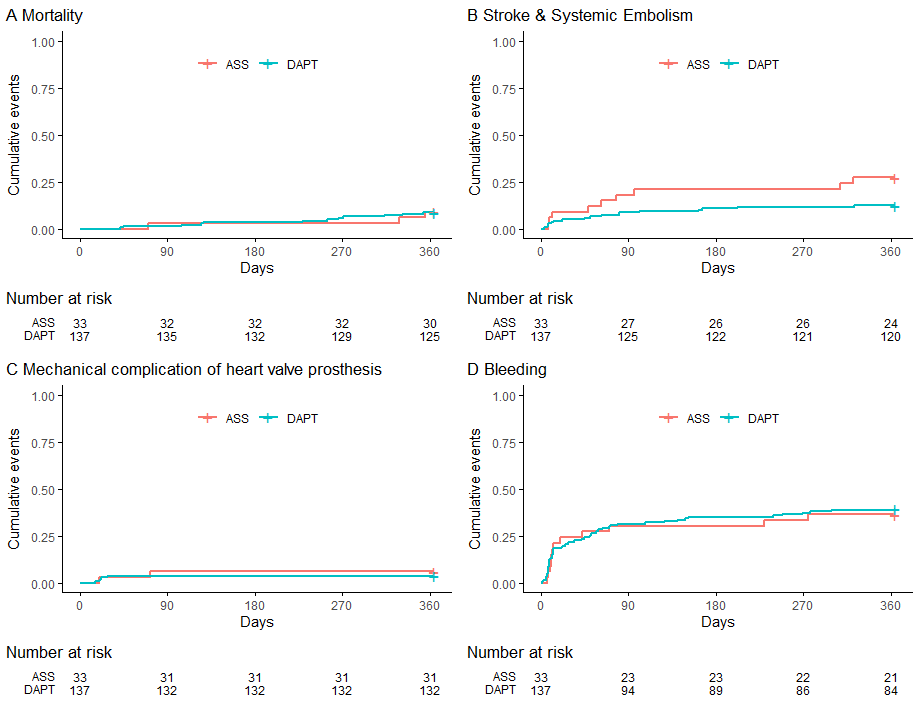


**Supplementary Figure S3** Kaplan-Meier-plots of cumulative incidence of (A) all-cause mortality, (B) stroke and/or systemic embolism, (C) mechanical complication of heart valve prosthesis and (D) bleeding for the subgroup analysis of ASS mono vs. ASS + Clopidogrel

*
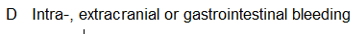
*
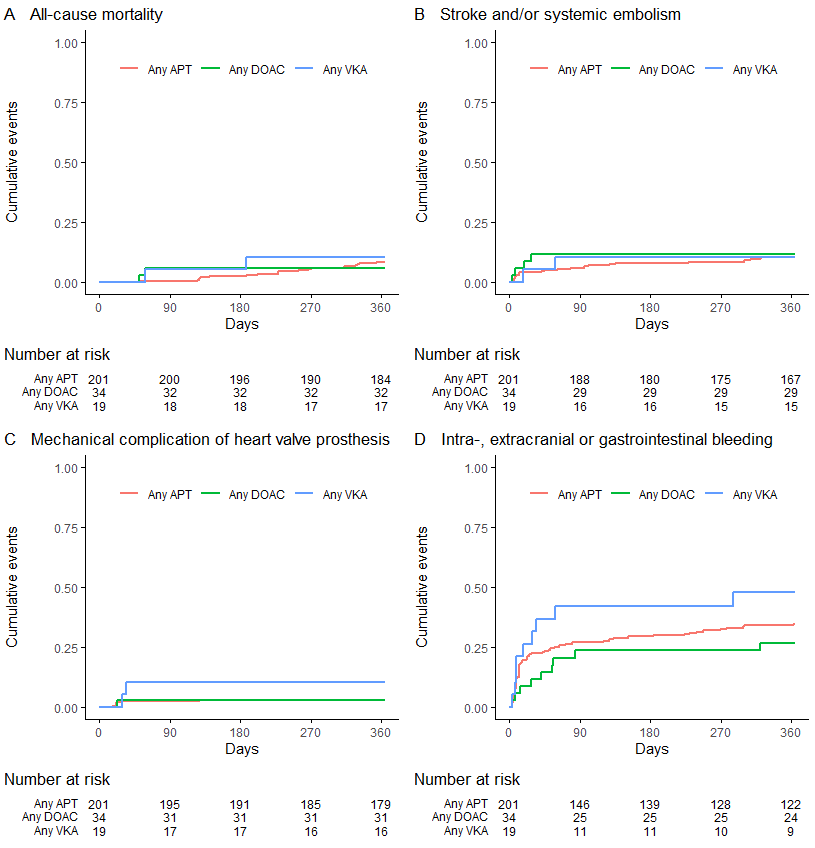


**Supplementary Figure S4** Kaplan-Meier-plots of cumulative incidence of all-cause mortality (A), stroke and/or systemic embolism (B), mechanical complication of heart valve prosthesis (C) and intra-, extra- or gastrointestinal bleeding (D) for patients without an indication for oral anticoagulation


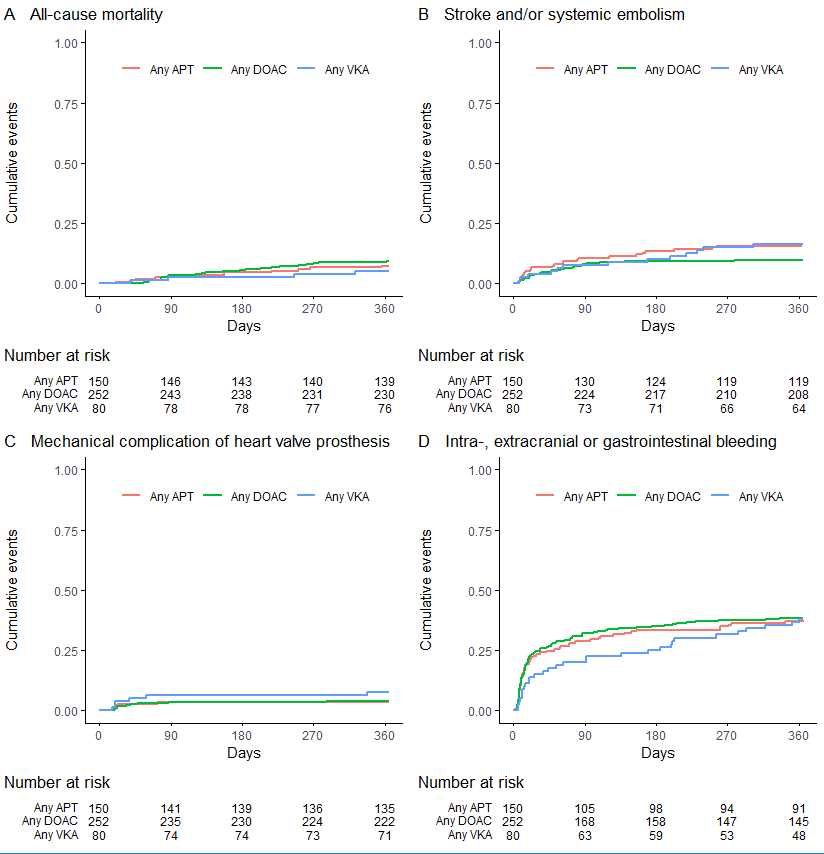


**Supplementary Figure S5** Kaplan-Meier-plots of cumulative incidence of all-cause mortality (A), stroke and/or systemic embolism (B), mechanical complication of heart valve prosthesis (C) and intra-, extra- or gastrointestinal bleeding (D) for patients with an indication for oral anticoagulation
